# Crystal structure of tetra­kis­(μ_2_-(*E*)-2,4-di­bromo-6-{[2-(pyridin-2-yl)eth­yl]imino­meth­yl}phen­olato)trizinc bis­(perchlorate) aceto­nitrile disolvate

**DOI:** 10.1107/S2056989018012100

**Published:** 2018-08-31

**Authors:** Ugochukwu Okeke, Raymond Otchere, Yilma Gultneh, Ray J. Butcher

**Affiliations:** a Department of Chemistry, Howard University, 525 College Street NW, Washington, DC 20059, USA

**Keywords:** crystal structure, Schiff base ligand, trinuclear Zn complex, halogen inter­actions

## Abstract

The title compound, [Zn_3_(C_14_H_11_Br_2_N_2_O)_4_](ClO_4_)_2_·2CH_3_CN, crystallizes as a symmetrical trinuclear cation with all three metal atoms being located on a twofold rotation axis. It contains a tetra­hedral Zn^II^ atom that bridges two six-coordinate Zn^II^ atoms. The complex contains N- and O-donor atoms of four tridentate 2,4-di­bromo-6-{[2-(pyridin-2-yl)eth­yl]imino­meth­yl}phenolate ligands. The ratio of Zn^II^ atoms to ligands is 3:4. The two terminal Zn^II^ cations adopt distorted octa­hedral geometries and the central Zn^II^ cation adopts a distorted tetra­hedral geometry. In the cation there are π–π inter­actions between the di­bromo­phenyl rings, as well as halogen-bonding inter­actions between the di­bromo­phenyl rings in the cation, which stabilize its conformation. In addition, there are C—H⋯O inter­actions between the anions and both the cations and solvent mol­ecules as well as C—H⋯N inter­actions between the cation and solvent mol­ecules. These inter­species inter­actions link the cations, anions and solvent mol­ecules into a complex three-dimensional array

## Abstract 2

The structure is reported of a complex containing a trinuclear Zn cation lying on a crystallographic twofold axis. It consists of a tetra­hedral Zn^II^ atom bridging two six-coordinate Zn^II^ atoms in which the two terminal Zn^II^ cations adopt distorted octa­hedral geometries and the central Zn^II^ cation adopts a distorted tetra­hedral geometry.

## Chemical context   

Zinc(II)-derived metalloenzymes are among the most common found in biology. Some enzymes containing zinc(II) include carbonic anhydrase, carb­oxy­peptidase, and phosphatase (Bertini *et al.*, 1994 ▸; McCall *et al.*, 2000 ▸). It is of inter­est to study zinc(II) complexes derived from tridentate Schiff base ligands because of the possibility of forming stable complex structures. Zinc(II) plays a structural role not only in enzymes but much progress has been made to incorporate it into metal–organic frameworks for drug storage and release, luminescence studies, and hydrogen-storage applications (An *et al.*, 2009 ▸; Bauer *et al.*, 2007 ▸; Rosi *et al.*, 2003 ▸).
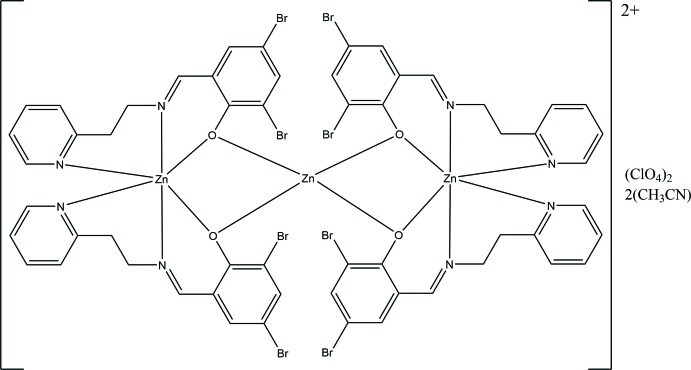


**Figure 1:**
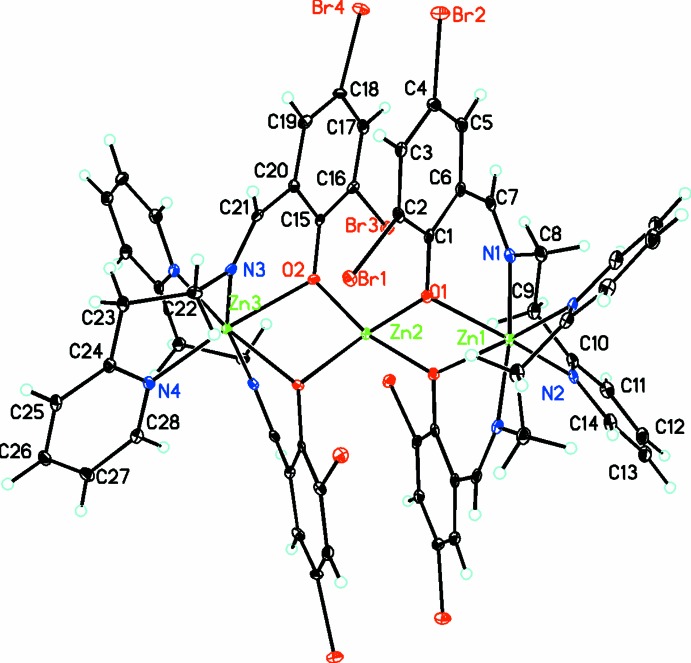
Diagram of the cation, tetra­kis­(μ_2_-(*E*)-2,4-di­bromo-6-({[2-(pyridin-2-yl)eth­yl]imino­meth­yl}phenolato)trizinc, showing the parallel di­bromo­phenyl rings. Atomic displacement parameters are at the 30% probability level.

Related complexes have been studied for their photoluminescent properties (Kundu *et al.*, 2015 ▸; Chakraborty *et al.*, 2013 ▸), drug therapeutic activity in DNA cleavage (Kumar *et al.*, 2011 ▸), and phosphatase mimetic activity (Kumar *et al.*, 2011 ▸; Gultneh *et al.*, 1999 ▸). The coordination environment of the title compound, illustrated in Fig. 1, has been observed in zinc(II) complexes with tridentate *N*,*N*,*O* ligands (Hens & Rajak, 2015 ▸; Kim *et al.*, 2015 ▸). Transition metal complexes of the related tridentate ligand, 1,3(2-pyridyl­imino­meth­yl)phenyl­enedi­amine, have been shown to form a variety of inter­esting complex structures (Kundu *et al.*, 2015 ▸; Kumar *et al.*, 2011 ▸; Bluhm *et al.*, 2003 ▸; Souza *et al.*, 2011 ▸; Sanyal *et al.*, 2014 ▸; Okeke *et al.*, 2017*a* ▸,*b* ▸; Okeke *et al.*, 2018 ▸). The presence of a substituent on the aromatic group may change the geometry, coordination number, and consequently the reactivity of the resulting complexes especially because of its location on the aromatic ring that coordinates to the metal ion through the phenoxide oxygen atom.

In a continuation of our model studies of zinc complexes as Lewis acid center in zinc-containing hydrolytic enzymes (Gultneh *et al.*, 1996 ▸; Gultneh *et al.*, 1999 ▸; Okeke *et al.*, 2017*a* ▸,*b* ▸) we report the structure of the title compound. This trinuclear zinc(II) complex has a 3:4 metal ion-to-ligand ratio. Since the title compound lies on a crystallographic twofold axis, the three zinc(II) ions form an angle of 180^o^ and thus are strictly linear. The central zinc atom is four coordinate and may serve as a suitable complex for various reactions because the Zn^II^ Lewis acid metal center contains vacant coordination sites for coordination to a nucleophile.

## Structural commentary   

The crystal structure of the title compound, [Zn_3_(C_14_H_11_Br_2_N_2_O)_4_](ClO_4_)_2_·2CH_3_CN, **1**, contains a complex cation as well as perchlorate anions and aceto­nitrile solvent mol­ecules and thus has an overall stoichiometry of [Zn_3_(*L*)_4_](ClO_4_)_2_ **^.^**2CH_3_CN where *L* is 2,4-di­bromo-6-{[(2-(pyridin-2-yl)eth­yl]imino­meth­yl}phenolate. The compound crystallizes in the monoclinic space group *C*2/*c* and the cation consists of the four equivalent *L* ligands, uniformly coordin­ated to three Zn^II^ cations.

The trinuclear complex cation, [Zn_3_(*L*)_4_]^2+^, lies on a crystallographic twofold axis (Fig. 1 ▸). The zinc(II) ions contain varying coordination spheres. Zn1 and Zn3 adopt O_2_N_4_ coordination spheres while the central zinc atom Zn2 adopts an O_4_ coordination sphere with a distorted tetra­hedral geometry with O—Zn—O bond angles ranging from 88.95 (11) to 120.11 (8)° and Zn—O bond lengths of 1.9512 (19) and 1.9602 (19) Å. For the six-coordinate terminal zinc atoms, as is usual for complexes containing both Schiff base imine and pyridine N donors, the former form shorter bonds [Zn1—N1 = 2.122 (2) Å and Zn3—-N3 = 2.067 (2) Å] while the latter form longer bonds [Zn1—N2 = 2.148 (2) Å and Zn3—N4 = 2.177 (2) Å] to zinc. The metrical parameters involving the bridging phenolate O donors are significantly different. The bonds to the central Zn2 are considerably shorter than those to the terminal Zn1 and Zn3 [O1—Zn1 = 2.194 (2) Å; O2—Zn3 = 2.266 (2) Å; O1—Zn2 = 1.960 (2) Å; O2—Zn2 = 1.951 (2) Å] and the bridging angles are Zn1—O1—Zn2 = 96.78 (8)° and Zn2—O2—Zn3 = 93.73 (8)°. The distortion from an octa­hedral geometry can be seen from the *cis* and *trans* angles which range from 77.49 (10) to 98.19 (9)° and 160.47 (13) to 173.41 (12)°, respectively. Since all three Zn atoms lie on the twofold axis, the Zn1—Zn2—Zn3 bond angle is exactly 180°. These metrical parameters are similar to those found in the most closely similar complex (Kim *et al.*, 2015 ▸) where Zn—O distances for the terminal Zn atoms range from 2.126 (3) to 2.155 (4) Å while those for the central Zn atom range from 1.945 (3) to 1.965 (4) Å with Zn—O—Zn bridging angles ranging from 97.3 (1) to 98.7 (1)°. The Zn—N_imine_ and Zn—N_py_ bond lengths range from 2.077 (4) to 2.117 (4) Å and 2.140 (4) to 2.176 (4) Å, respectively. In this complex there is no crystallographically imposed symmetry; however, the Zn—Zn—Zn bond angle is still close to 180 at 172.51 (3)°.

## Supra­molecular features   

**Figure 2:**
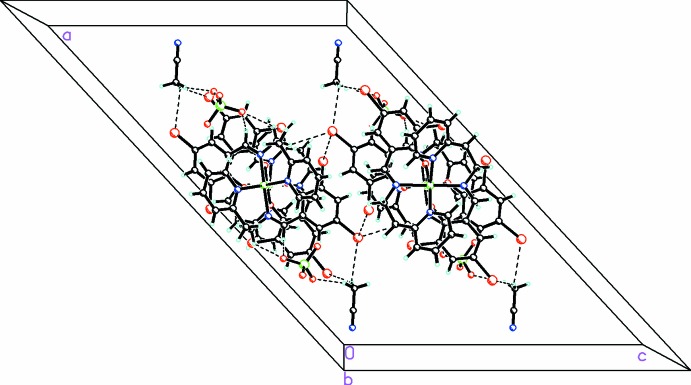
Packing diagram viewed along the *b* axis showing the extensive C—H⋯O, C—H⋯N, and C—H⋯Br inter­actions (shown as dashed lines) linking the cations, anions, and solvent mol­ecules into a complex three-dimensional array.

In the cation there are π–π inter­actions between the di­bromo­phenyl rings [centroid–centroid distance = 3.602 (2) Å; *CgI*⋯perp = 3.344 (1) Å; slippage = 1.319 (2) Å] as well as halogen-bonding inter­actions [Br⋯Br 3.6123 (5) Å; C—Br⋯Br, 129.08 (9)°] between the di­bromo­phenyl rings in the cation, which stabilize its conformation. In addition there C–H⋯O inter­actions between the anions and both the cations and solvent mol­ecules as well as C—H⋯N inter­actions between the cation and solvent mol­ecules (Table 1 ▸). These inter­species inter­actions link the cations, anions and solvent mol­ecules into a complex three-dimensional array as shown in Fig. 2 ▸.

## Database survey   

A search of the Cambridge Structural Database for complexes of zinc coordinated to (*E*)-2-({[2-(pyridin-2-yl)eth­yl]imino}­meth­yl)phenolato type ligands gave 26 hits of which only one was similar to the title compound in that it contained a trinuclear Zn complex where this ligand was acting as a bridging group to the central Zn atom (Diop *et al.*, 2014 ▸) . However, in this case each terminal Zn complex only provided one bridging O atom and the coordination sphere of the central Zn was hexa­coordinate with six O-atom donors in contrast to the title compound where the central Zn is four–coordinate with the terminal Zn complexes provided two bridging atoms through their phenolic O atoms. A search for structures containing three zinc atoms with the central zinc atom in an μ_2_-*O*_4_ environment and with the terminal zinc atoms coordinated to Schiff base derivatives gave four hits [MAYVEQ, Quilter *et al.*, 2017 ▸; GOWGUW, Hens & Rajak, 2015 ▸; HUQVUL, Akine *et al.*, 2009 ▸; KURPAL, Kim *et al.*, 2015 ▸] of which that using the ligand, 2-methyl-6-{[(pyridin-2-ylmeth­yl)imino]­meth­yl}phenol in the presence of NH_4_PF_6_ resulted in a closely related trinuclear zinc complex with the central Zn atom four-coordinate with only O-atom donors from the bridging phenolate ligands (Kim *et al.*, 2015 ▸). The major differences between this complex and **1** is a –CH_2_– link between the imine N and pyridine ring in the former instead of a –CH_2_-CH_2_– link in the latter, and different substituents on the phenyl ring.

## Synthesis and crystallization   

2-(2-Pyrid­yl)ethyl­amine (0.3023 g, 2.474 mmol) was dissolved in 50 mL of methanol. 3,5-Di­bromo­salicyl­aldehyde (0.6927 g, 2.474 mmol) was added to the solution and the mixture was refluxed for 5 h. The zinc(II) complex was prepared by reacting the ligand in 50 ml of methanol with Zn(ClO_4_)_2_·6H_2_O (1.3821 g, 3.712 mmol) with no added base. The mixture was stirred at room temperature overnight. The methanol was removed by rotary evaporation. The product was crystallized by slow evaporation of a solution in acetonitrile giving pale-yellow to colorless crystals.

## Refinement   

Crystal data, data collection and structure refinement details are summarized in Table 2 ▸. All hydrogen atoms were refined using a riding model with C—H distances of 0.95 to 0.99 Å and *U*_iso_(H) = 1.2*U*_eq_(C) or 1.5*U*_eq_(CH_3_).

## Supplementary Material

Crystal structure: contains datablock(s) I. DOI: 10.1107/S2056989018012100/jj2202sup1.cif

Structure factors: contains datablock(s) I. DOI: 10.1107/S2056989018012100/jj2202Isup2.hkl

CCDC reference: 1863971

Additional supporting information:  crystallographic information; 3D view; checkCIF report

## Figures and Tables

**Table 1:** Hydrogen-bond geometry (Å, °)

*D*—H⋯*A*	*D*—H	H⋯*A*	*D*⋯*A*	*D*—H⋯*A*
C9—H9*A*⋯Br1^i^	0.99	2.92	3.854 (3)	157
C9—H9*A*⋯O1^i^	0.99	2.60	3.317 (4)	129
C21—H21*A*⋯O14	0.95	2.57	3.080 (4)	114
C22—H22*A*⋯O14	0.99	2.58	3.099 (4)	113
C22—H22*B*⋯Br4^ii^	0.99	2.96	3.664 (3)	129
C23—H23*A*⋯O12^iii^	0.99	2.58	3.427 (4)	144
C28—H28*A*⋯N3^i^	0.95	2.60	3.236 (4)	125
C2*S*—H2*S*1⋯O11	0.98	2.60	3.556 (4)	165
C2*S*—H2*S*2⋯Br2	0.98	3.02	3.935 (4)	157
C2*S*—H2*S*2⋯Br4	0.98	3.04	3.561 (3)	115

Symmetry codes: (i) 
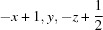
; (ii) 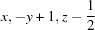; (iii) 
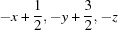
.

**Table 2:** Experimental details

Crystal data	
Chemical formula	[Zn_3_(C_14_H_11_Br_2_N_2_O)_4_](ClO_4_)_2_·2C_2_H_3_N
*M*_r_	2009.39
Crystal system, space group	Monoclinic, *C*2/*c*
Temperature (K)	100
*a*, *b*, *c* (Å)	30.797 (3), 13.8527 (12), 21.135 (3)
β (°)	132.857 (1)
*V* (Å^3^)	6609.6 (13)
*Z*	4
Radiation type	Mo *K*α
μ (mm^−1^)	6.07
Crystal size (mm)	0.35 × 0.31 × 0.24

Data collection	
Diffractometer	Bruker APEXII CCD
Absorption correction	Multi-scan (*SADABS*; Sheldrick, 1996 ▸)
*T*_min_, *T*_max_	0.585, 0.746
No. of measured, independent and observed [*I* > 2σ(*I*)] reflections	23422, 7310, 5978
*R*_int_	0.042
(sin θ/λ)_max_ (Å^−1^)	0.643

Refinement	
*R*[*F* ^2^ > 2σ(*F* ^2^)], *wR*(*F* ^2^), *S*	0.028, 0.066, 1.01
No. of reflections	7310
No. of parameters	431
H-atom treatment	H-atom parameters constrained
Δρ_max_, Δρ_min_ (e Å^−3^)	0.52, −0.56

Computer programs: *APEX2* and *SAINT* (Bruker, 2014 ▸), *SHELXT* (Sheldrick, 2015*a* ▸), *SHELXL2018* (Sheldrick, 2015*b* ▸) and *SHELXTL* (Sheldrick, 2008 ▸).

## supplementary crystallographic information

### Crystal data

**Table:**

[Zn_3_(C_14_H_11_Br_2_N_2_O)_4_](ClO_4_)_2_·2C_2_H_3_N	*F*(000) = 3920
*M**_r_* = 2009.39	*D*_x_ = 2.019 Mg m^−^^3^
Monoclinic, *C*2/*c*	Mo *K*α radiation, λ = 0.71073 Å
*a* = 30.797 (3) Å	Cell parameters from 6020 reflections
*b* = 13.8527 (12) Å	θ = 2.5–27.1°
*c* = 21.135 (3) Å	µ = 6.07 mm^−^^1^
β = 132.857 (1)°	*T* = 100 K
*V* = 6609.6 (13) Å^3^	Chunk, colorless
*Z* = 4	0.35 × 0.31 × 0.24 mm

### Data collection

**Table:**

Bruker APEXII CCD diffractometer	5978 reflections with *I* > 2σ(*I*)
φ and ω scans	*R*_int_ = 0.042
Absorption correction: multi-scan (SADABS; Sheldrick, 1996)	θ_max_ = 27.2°, θ_min_ = 1.7°
*T*_min_ = 0.585, *T*_max_ = 0.746	*h* = −37→39
23422 measured reflections	*k* = −17→17
7310 independent reflections	*l* = −27→25

### Refinement

**Table:**

Refinement on *F*^2^	0 restraints
Least-squares matrix: full	Hydrogen site location: inferred from neighbouring sites
*R*[*F*^2^ > 2σ(*F*^2^)] = 0.028	H-atom parameters constrained
*wR*(*F*^2^) = 0.066	*w* = 1/[σ^2^(*F*_o_^2^) + (0.0285*P*)^2^] where *P* = (*F*_o_^2^ + 2*F*_c_^2^)/3
*S* = 1.01	(Δ/σ)_max_ = 0.001
7310 reflections	Δρ_max_ = 0.52 e Å^−^^3^
431 parameters	Δρ_min_ = −0.56 e Å^−^^3^

### Special details

**Table:**

Geometry. All esds (except the esd in the dihedral angle between two l.s. planes) are estimated using the full covariance matrix. The cell esds are taken into account individually in the estimation of esds in distances, angles and torsion angles; correlations between esds in cell parameters are only used when they are defined by crystal symmetry. An approximate (isotropic) treatment of cell esds is used for estimating esds involving l.s. planes.

### Fractional atomic coordinates and isotropic or equivalent isotropic displacement parameters (Å^2^)

**Table:**

	*x*	*y*	*z*	*U*_iso_*/*U*_eq_	
Zn1	0.500000	0.14227 (3)	0.250000	0.01098 (11)	
Zn2	0.500000	0.36678 (3)	0.250000	0.00984 (10)	
Zn3	0.500000	0.58950 (3)	0.250000	0.00943 (10)	
Br1	0.32810 (2)	0.35929 (2)	0.03320 (2)	0.01421 (7)	
Br2	0.23309 (2)	0.24841 (2)	0.17654 (2)	0.01768 (8)	
Br3	0.57097 (2)	0.36780 (2)	0.49927 (2)	0.01481 (7)	
Br4	0.34475 (2)	0.39606 (2)	0.38605 (2)	0.01633 (8)	
O1	0.43973 (8)	0.26581 (13)	0.19910 (12)	0.0116 (4)	
O2	0.50660 (8)	0.46259 (13)	0.32385 (12)	0.0105 (4)	
N1	0.49899 (10)	0.15108 (16)	0.34924 (15)	0.0120 (5)	
N2	0.57350 (10)	0.04396 (16)	0.32655 (16)	0.0128 (5)	
N3	0.41056 (10)	0.56419 (16)	0.17387 (14)	0.0100 (5)	
N4	0.48785 (10)	0.69446 (17)	0.16238 (15)	0.0120 (5)	
C1	0.39443 (13)	0.26316 (19)	0.19470 (18)	0.0115 (6)	
C2	0.33828 (12)	0.3003 (2)	0.12332 (18)	0.0125 (6)	
C3	0.29034 (12)	0.2968 (2)	0.11669 (18)	0.0125 (6)	
H3A	0.252883	0.321421	0.066928	0.015*	
C4	0.29783 (13)	0.2567 (2)	0.18389 (19)	0.0134 (6)	
C5	0.35244 (12)	0.2226 (2)	0.25680 (19)	0.0133 (6)	
H5A	0.357743	0.198681	0.303803	0.016*	
C6	0.40025 (13)	0.2231 (2)	0.26161 (19)	0.0125 (6)	
C7	0.45614 (12)	0.1826 (2)	0.34060 (19)	0.0130 (6)	
H7A	0.460954	0.179563	0.389986	0.016*	
C8	0.54796 (13)	0.1062 (2)	0.43393 (19)	0.0165 (7)	
H8A	0.539244	0.036827	0.431509	0.020*	
H8B	0.550395	0.136791	0.478608	0.020*	
C9	0.60749 (13)	0.1166 (2)	0.45962 (19)	0.0161 (7)	
H9A	0.611367	0.183690	0.447945	0.019*	
H9B	0.639425	0.105514	0.522545	0.019*	
C10	0.61651 (13)	0.0488 (2)	0.41373 (19)	0.0146 (6)	
C11	0.66718 (13)	−0.0086 (2)	0.45956 (19)	0.0172 (7)	
H11A	0.696529	−0.005330	0.520794	0.021*	
C12	0.67487 (14)	−0.0699 (2)	0.4164 (2)	0.0200 (7)	
H12A	0.709631	−0.108159	0.447257	0.024*	
C13	0.63092 (14)	−0.0745 (2)	0.3273 (2)	0.0191 (7)	
H13A	0.634965	−0.115895	0.295756	0.023*	
C14	0.58105 (13)	−0.0179 (2)	0.2852 (2)	0.0176 (7)	
H14A	0.550541	−0.022584	0.224201	0.021*	
C15	0.47154 (12)	0.45477 (19)	0.33930 (18)	0.0104 (6)	
C16	0.49187 (12)	0.4129 (2)	0.41645 (18)	0.0112 (6)	
C17	0.45533 (12)	0.3990 (2)	0.43196 (18)	0.0131 (6)	
H17A	0.470451	0.370516	0.484520	0.016*	
C18	0.39659 (13)	0.4268 (2)	0.37030 (19)	0.0123 (6)	
C19	0.37482 (13)	0.4694 (2)	0.29449 (18)	0.0130 (6)	
H19A	0.334411	0.488362	0.252538	0.016*	
C20	0.41171 (12)	0.4850 (2)	0.27879 (18)	0.0111 (6)	
C21	0.38403 (12)	0.52587 (19)	0.19498 (18)	0.0098 (6)	
H21A	0.342005	0.523861	0.151561	0.012*	
C22	0.37350 (12)	0.6000 (2)	0.08515 (18)	0.0124 (6)	
H22A	0.331359	0.586806	0.053059	0.015*	
H22B	0.383766	0.565070	0.055952	0.015*	
C23	0.38206 (13)	0.7089 (2)	0.08335 (19)	0.0145 (6)	
H23A	0.346350	0.734522	0.026604	0.017*	
H23B	0.384761	0.740418	0.127954	0.017*	
C24	0.43538 (13)	0.7373 (2)	0.09813 (19)	0.0129 (6)	
C25	0.42998 (13)	0.8065 (2)	0.04501 (19)	0.0148 (6)	
H25A	0.392689	0.835759	−0.000033	0.018*	
C26	0.47876 (13)	0.8322 (2)	0.05796 (19)	0.0153 (6)	
H26A	0.475620	0.879928	0.022729	0.018*	
C27	0.53280 (13)	0.7871 (2)	0.12365 (19)	0.0156 (6)	
H27A	0.567076	0.802490	0.133565	0.019*	
C28	0.53512 (13)	0.7196 (2)	0.17379 (19)	0.0145 (6)	
H28A	0.571971	0.689164	0.218806	0.017*	
Cl1	0.27556 (3)	0.71818 (5)	0.15347 (5)	0.01814 (16)	
O11	0.23291 (11)	0.65110 (17)	0.13573 (16)	0.0326 (6)	
O12	0.25024 (10)	0.81314 (16)	0.12595 (16)	0.0285 (6)	
O13	0.32715 (10)	0.7195 (2)	0.24425 (15)	0.0384 (7)	
O14	0.29229 (12)	0.69039 (18)	0.10698 (18)	0.0383 (7)	
N1S	0.08476 (12)	0.49951 (19)	0.11043 (17)	0.0250 (7)	
C1S	0.13487 (15)	0.4960 (2)	0.1626 (2)	0.0208 (7)	
C2S	0.19918 (14)	0.4933 (3)	0.2300 (2)	0.0327 (9)	
H2S1	0.215955	0.538085	0.215527	0.049*	
H2S2	0.213104	0.427731	0.234893	0.049*	
H2S3	0.211812	0.512395	0.285114	0.049*	

### Atomic displacement parameters (Å^2^)

**Table:**

	*U*^11^	*U*^22^	*U*^33^	*U*^12^	*U*^13^	*U*^23^
Zn1	0.0127 (2)	0.0081 (2)	0.0110 (2)	0.000	0.0076 (2)	0.000
Zn2	0.0117 (2)	0.0072 (2)	0.0126 (2)	0.000	0.0091 (2)	0.000
Zn3	0.0097 (2)	0.0082 (2)	0.0103 (2)	0.000	0.0068 (2)	0.000
Br1	0.01578 (15)	0.01304 (15)	0.01356 (14)	−0.00108 (12)	0.00988 (13)	0.00087 (12)
Br2	0.01496 (15)	0.02397 (17)	0.01785 (15)	−0.00013 (12)	0.01263 (14)	−0.00039 (13)
Br3	0.01271 (14)	0.01568 (15)	0.01417 (15)	0.00158 (12)	0.00840 (13)	0.00163 (12)
Br4	0.01600 (15)	0.02204 (16)	0.01645 (15)	−0.00221 (12)	0.01321 (13)	0.00029 (13)
O1	0.0112 (10)	0.0095 (10)	0.0142 (10)	−0.0017 (8)	0.0087 (9)	−0.0025 (8)
O2	0.0122 (10)	0.0106 (10)	0.0126 (10)	−0.0008 (8)	0.0100 (9)	−0.0022 (8)
N1	0.0125 (12)	0.0092 (12)	0.0115 (12)	−0.0006 (10)	0.0070 (11)	0.0016 (10)
N2	0.0160 (12)	0.0068 (12)	0.0155 (12)	−0.0018 (10)	0.0107 (11)	0.0007 (10)
N3	0.0119 (12)	0.0090 (12)	0.0100 (11)	0.0011 (10)	0.0079 (10)	−0.0010 (10)
N4	0.0135 (12)	0.0096 (12)	0.0134 (12)	−0.0011 (10)	0.0093 (11)	−0.0005 (10)
C1	0.0139 (14)	0.0057 (13)	0.0140 (14)	−0.0007 (11)	0.0092 (13)	−0.0025 (11)
C2	0.0162 (15)	0.0079 (14)	0.0133 (14)	−0.0048 (12)	0.0099 (13)	−0.0029 (12)
C3	0.0129 (14)	0.0083 (14)	0.0144 (14)	0.0008 (11)	0.0086 (13)	0.0003 (12)
C4	0.0135 (14)	0.0126 (15)	0.0174 (15)	−0.0033 (12)	0.0118 (13)	−0.0028 (12)
C5	0.0151 (15)	0.0103 (14)	0.0148 (15)	−0.0023 (12)	0.0104 (13)	−0.0018 (12)
C6	0.0151 (15)	0.0059 (13)	0.0157 (15)	−0.0012 (11)	0.0102 (13)	−0.0020 (12)
C7	0.0167 (15)	0.0074 (14)	0.0145 (14)	−0.0052 (12)	0.0104 (13)	−0.0035 (12)
C8	0.0195 (16)	0.0128 (15)	0.0157 (15)	0.0013 (13)	0.0113 (14)	0.0029 (13)
C9	0.0156 (15)	0.0131 (15)	0.0122 (14)	0.0005 (12)	0.0066 (13)	0.0007 (12)
C10	0.0164 (15)	0.0113 (15)	0.0148 (15)	−0.0024 (12)	0.0100 (13)	0.0008 (12)
C11	0.0128 (15)	0.0170 (16)	0.0136 (15)	−0.0003 (12)	0.0057 (13)	0.0023 (13)
C12	0.0181 (16)	0.0130 (15)	0.0246 (17)	0.0033 (13)	0.0127 (15)	0.0041 (14)
C13	0.0238 (17)	0.0125 (15)	0.0221 (17)	0.0014 (13)	0.0160 (15)	0.0009 (13)
C14	0.0192 (16)	0.0120 (15)	0.0152 (15)	−0.0014 (13)	0.0092 (14)	0.0006 (13)
C15	0.0143 (14)	0.0046 (13)	0.0163 (14)	−0.0018 (11)	0.0120 (13)	−0.0037 (11)
C16	0.0096 (14)	0.0110 (14)	0.0113 (14)	0.0008 (11)	0.0065 (12)	−0.0020 (12)
C17	0.0175 (15)	0.0096 (14)	0.0119 (14)	−0.0025 (12)	0.0099 (13)	−0.0015 (12)
C18	0.0137 (14)	0.0132 (15)	0.0168 (15)	−0.0026 (12)	0.0130 (13)	−0.0030 (12)
C19	0.0137 (14)	0.0136 (15)	0.0110 (14)	−0.0001 (12)	0.0082 (12)	−0.0038 (12)
C20	0.0128 (14)	0.0082 (14)	0.0132 (14)	−0.0014 (11)	0.0092 (12)	−0.0014 (12)
C21	0.0080 (13)	0.0091 (14)	0.0114 (13)	0.0008 (11)	0.0062 (12)	−0.0020 (11)
C22	0.0109 (14)	0.0154 (15)	0.0110 (14)	−0.0017 (12)	0.0074 (12)	−0.0013 (12)
C23	0.0154 (15)	0.0120 (15)	0.0146 (15)	0.0041 (12)	0.0096 (13)	0.0054 (12)
C24	0.0170 (15)	0.0085 (14)	0.0142 (14)	−0.0024 (12)	0.0110 (13)	−0.0033 (12)
C25	0.0168 (15)	0.0101 (14)	0.0154 (15)	0.0027 (12)	0.0102 (13)	0.0034 (12)
C26	0.0242 (16)	0.0113 (14)	0.0147 (15)	−0.0012 (13)	0.0150 (14)	0.0003 (12)
C27	0.0181 (15)	0.0147 (15)	0.0171 (15)	−0.0045 (13)	0.0133 (14)	−0.0016 (13)
C28	0.0147 (15)	0.0129 (15)	0.0150 (15)	0.0011 (12)	0.0097 (13)	0.0002 (12)
Cl1	0.0169 (4)	0.0209 (4)	0.0201 (4)	0.0014 (3)	0.0140 (3)	−0.0002 (3)
O11	0.0386 (15)	0.0322 (14)	0.0322 (14)	−0.0188 (12)	0.0262 (13)	−0.0101 (12)
O12	0.0306 (13)	0.0187 (12)	0.0425 (15)	0.0058 (10)	0.0273 (13)	−0.0003 (11)
O13	0.0182 (13)	0.0659 (19)	0.0193 (13)	−0.0026 (13)	0.0080 (11)	0.0072 (13)
O14	0.0582 (17)	0.0329 (15)	0.0568 (18)	0.0171 (13)	0.0521 (16)	0.0089 (13)
N1S	0.0239 (16)	0.0186 (15)	0.0221 (15)	−0.0001 (12)	0.0115 (14)	−0.0009 (12)
C1S	0.0312 (19)	0.0096 (15)	0.0246 (18)	0.0020 (14)	0.0202 (17)	0.0011 (13)
C2S	0.0239 (19)	0.0217 (18)	0.042 (2)	0.0014 (15)	0.0182 (18)	0.0031 (17)

### Geometric parameters (Å, º)

**Table:**

Zn1—N1^i^	2.122 (2)	C9—C10	1.505 (4)
Zn1—N1	2.122 (2)	C9—H9A	0.9900
Zn1—N2^i^	2.148 (2)	C9—H9B	0.9900
Zn1—N2	2.148 (2)	C10—C11	1.396 (4)
Zn1—O1^i^	2.1943 (19)	C11—C12	1.379 (4)
Zn1—O1	2.1943 (19)	C11—H11A	0.9500
Zn1—Zn2	3.1100 (7)	C12—C13	1.384 (4)
Zn2—O2	1.9512 (19)	C12—H12A	0.9500
Zn2—O2^i^	1.9512 (19)	C13—C14	1.381 (4)
Zn2—O1	1.9602 (19)	C13—H13A	0.9500
Zn2—O1^i^	1.9602 (19)	C14—H14A	0.9500
Zn2—Zn3	3.0852 (7)	C15—C16	1.414 (4)
Zn3—N3	2.067 (2)	C15—C20	1.415 (4)
Zn3—N3^i^	2.067 (2)	C16—C17	1.383 (4)
Zn3—N4^i^	2.177 (2)	C17—C18	1.383 (4)
Zn3—N4	2.177 (2)	C17—H17A	0.9500
Zn3—O2	2.2664 (19)	C18—C19	1.380 (4)
Zn3—O2^i^	2.2664 (19)	C19—C20	1.400 (4)
Br1—C2	1.892 (3)	C19—H19A	0.9500
Br2—C4	1.895 (3)	C20—C21	1.460 (4)
Br3—C16	1.894 (3)	C21—H21A	0.9500
Br4—C18	1.888 (3)	C22—C23	1.536 (4)
O1—C1	1.334 (3)	C22—H22A	0.9900
O2—C15	1.328 (3)	C22—H22B	0.9900
N1—C7	1.280 (4)	C23—C24	1.500 (4)
N1—C8	1.481 (4)	C23—H23A	0.9900
N2—C14	1.351 (4)	C23—H23B	0.9900
N2—C10	1.354 (4)	C24—C25	1.398 (4)
N3—C21	1.282 (4)	C25—C26	1.378 (4)
N3—C22	1.467 (3)	C25—H25A	0.9500
N4—C28	1.350 (4)	C26—C27	1.395 (4)
N4—C24	1.350 (4)	C26—H26A	0.9500
C1—C2	1.408 (4)	C27—C28	1.378 (4)
C1—C6	1.412 (4)	C27—H27A	0.9500
C2—C3	1.386 (4)	C28—H28A	0.9500
C3—C4	1.390 (4)	Cl1—O13	1.435 (2)
C3—H3A	0.9500	Cl1—O11	1.435 (2)
C4—C5	1.379 (4)	Cl1—O12	1.435 (2)
C5—C6	1.405 (4)	Cl1—O14	1.438 (2)
C5—H5A	0.9500	N1S—C1S	1.134 (4)
C6—C7	1.468 (4)	C1S—C2S	1.454 (5)
C7—H7A	0.9500	C2S—H2S1	0.9800
C8—C9	1.524 (4)	C2S—H2S2	0.9800
C8—H8A	0.9900	C2S—H2S3	0.9800
C8—H8B	0.9900		
			
N1^i^—Zn1—N1	173.41 (12)	C1—C6—C7	122.7 (3)
N1^i^—Zn1—N2^i^	90.48 (9)	N1—C7—C6	126.2 (3)
N1—Zn1—N2^i^	93.70 (9)	N1—C7—H7A	116.9
N1^i^—Zn1—N2	93.70 (9)	C6—C7—H7A	116.9
N1—Zn1—N2	90.48 (9)	N1—C8—C9	112.4 (2)
N2^i^—Zn1—N2	101.31 (12)	N1—C8—H8A	109.1
N1^i^—Zn1—O1^i^	82.16 (8)	C9—C8—H8A	109.1
N1—Zn1—O1^i^	92.68 (8)	N1—C8—H8B	109.1
N2^i^—Zn1—O1^i^	166.02 (8)	C9—C8—H8B	109.1
N2—Zn1—O1^i^	91.06 (8)	H8A—C8—H8B	107.9
N1^i^—Zn1—O1	92.68 (8)	C10—C9—C8	114.6 (2)
N1—Zn1—O1	82.16 (8)	C10—C9—H9A	108.6
N2^i^—Zn1—O1	91.06 (8)	C8—C9—H9A	108.6
N2—Zn1—O1	166.02 (8)	C10—C9—H9B	108.6
O1^i^—Zn1—O1	77.49 (10)	C8—C9—H9B	108.6
N1^i^—Zn1—Zn2	86.70 (6)	H9A—C9—H9B	107.6
N1—Zn1—Zn2	86.70 (6)	N2—C10—C11	120.9 (3)
N2^i^—Zn1—Zn2	129.35 (6)	N2—C10—C9	118.0 (3)
N2—Zn1—Zn2	129.34 (6)	C11—C10—C9	121.1 (3)
O1^i^—Zn1—Zn2	38.75 (5)	C12—C11—C10	120.4 (3)
O1—Zn1—Zn2	38.75 (5)	C12—C11—H11A	119.8
O2—Zn2—O2^i^	94.29 (11)	C10—C11—H11A	119.8
O2—Zn2—O1	117.97 (8)	C11—C12—C13	118.6 (3)
O2^i^—Zn2—O1	120.11 (8)	C11—C12—H12A	120.7
O2—Zn2—O1^i^	120.11 (8)	C13—C12—H12A	120.7
O2^i^—Zn2—O1^i^	117.96 (8)	C14—C13—C12	118.7 (3)
O1—Zn2—O1^i^	88.95 (11)	C14—C13—H13A	120.7
O2—Zn2—Zn3	47.14 (6)	C12—C13—H13A	120.7
O2^i^—Zn2—Zn3	47.14 (6)	N2—C14—C13	123.3 (3)
O1—Zn2—Zn3	135.52 (6)	N2—C14—H14A	118.4
O1^i^—Zn2—Zn3	135.52 (6)	C13—C14—H14A	118.4
O2—Zn2—Zn1	132.86 (6)	O2—C15—C16	121.4 (2)
O2^i^—Zn2—Zn1	132.86 (6)	O2—C15—C20	122.1 (3)
O1—Zn2—Zn1	44.48 (6)	C16—C15—C20	116.5 (3)
O1^i^—Zn2—Zn1	44.48 (6)	C17—C16—C15	122.5 (3)
Zn3—Zn2—Zn1	180.0	C17—C16—Br3	118.5 (2)
N3—Zn3—N3^i^	160.47 (13)	C15—C16—Br3	118.9 (2)
N3—Zn3—N4^i^	98.19 (9)	C16—C17—C18	119.4 (3)
N3^i^—Zn3—N4^i^	94.83 (9)	C16—C17—H17A	120.3
N3—Zn3—N4	94.83 (9)	C18—C17—H17A	120.3
N3^i^—Zn3—N4	98.19 (9)	C19—C18—C17	120.3 (3)
N4^i^—Zn3—N4	96.20 (13)	C19—C18—Br4	120.0 (2)
N3—Zn3—O2	81.50 (8)	C17—C18—Br4	119.5 (2)
N3^i^—Zn3—O2	83.37 (8)	C18—C19—C20	120.6 (3)
N4^i^—Zn3—O2	92.83 (8)	C18—C19—H19A	119.7
N4—Zn3—O2	170.67 (8)	C20—C19—H19A	119.7
N3—Zn3—O2^i^	83.37 (8)	C19—C20—C15	120.6 (3)
N3^i^—Zn3—O2^i^	81.50 (8)	C19—C20—C21	116.7 (3)
N4^i^—Zn3—O2^i^	170.67 (8)	C15—C20—C21	122.6 (3)
N4—Zn3—O2^i^	92.83 (8)	N3—C21—C20	126.8 (3)
O2—Zn3—O2^i^	78.27 (10)	N3—C21—H21A	116.6
N3—Zn3—Zn2	80.23 (6)	C20—C21—H21A	116.6
N3^i^—Zn3—Zn2	80.23 (6)	N3—C22—C23	111.5 (2)
N4^i^—Zn3—Zn2	131.90 (6)	N3—C22—H22A	109.3
N4—Zn3—Zn2	131.90 (6)	C23—C22—H22A	109.3
O2—Zn3—Zn2	39.13 (5)	N3—C22—H22B	109.3
O2^i^—Zn3—Zn2	39.13 (5)	C23—C22—H22B	109.3
C1—O1—Zn2	127.79 (17)	H22A—C22—H22B	108.0
C1—O1—Zn1	120.56 (17)	C24—C23—C22	115.8 (2)
Zn2—O1—Zn1	96.78 (8)	C24—C23—H23A	108.3
C15—O2—Zn2	118.78 (16)	C22—C23—H23A	108.3
C15—O2—Zn3	121.70 (16)	C24—C23—H23B	108.3
Zn2—O2—Zn3	93.73 (8)	C22—C23—H23B	108.3
C7—N1—C8	114.8 (3)	H23A—C23—H23B	107.4
C7—N1—Zn1	125.8 (2)	N4—C24—C25	121.1 (3)
C8—N1—Zn1	118.89 (19)	N4—C24—C23	119.1 (3)
C14—N2—C10	118.1 (3)	C25—C24—C23	119.8 (3)
C14—N2—Zn1	118.13 (19)	C26—C25—C24	120.0 (3)
C10—N2—Zn1	123.2 (2)	C26—C25—H25A	120.0
C21—N3—C22	117.3 (2)	C24—C25—H25A	120.0
C21—N3—Zn3	128.81 (19)	C25—C26—C27	118.9 (3)
C22—N3—Zn3	113.82 (17)	C25—C26—H26A	120.5
C28—N4—C24	118.3 (3)	C27—C26—H26A	120.5
C28—N4—Zn3	118.68 (19)	C28—C27—C26	118.2 (3)
C24—N4—Zn3	123.00 (19)	C28—C27—H27A	120.9
O1—C1—C2	121.9 (3)	C26—C27—H27A	120.9
O1—C1—C6	121.8 (3)	N4—C28—C27	123.5 (3)
C2—C1—C6	116.3 (3)	N4—C28—H28A	118.2
C3—C2—C1	122.9 (3)	C27—C28—H28A	118.2
C3—C2—Br1	118.5 (2)	O13—Cl1—O11	110.02 (15)
C1—C2—Br1	118.6 (2)	O13—Cl1—O12	109.40 (16)
C2—C3—C4	119.1 (3)	O11—Cl1—O12	109.83 (15)
C2—C3—H3A	120.5	O13—Cl1—O14	109.36 (16)
C4—C3—H3A	120.5	O11—Cl1—O14	109.75 (16)
C5—C4—C3	120.5 (3)	O12—Cl1—O14	108.47 (15)
C5—C4—Br2	119.1 (2)	N1S—C1S—C2S	178.9 (4)
C3—C4—Br2	120.4 (2)	C1S—C2S—H2S1	109.5
C4—C5—C6	120.0 (3)	C1S—C2S—H2S2	109.5
C4—C5—H5A	120.0	H2S1—C2S—H2S2	109.5
C6—C5—H5A	120.0	C1S—C2S—H2S3	109.5
C5—C6—C1	121.1 (3)	H2S1—C2S—H2S3	109.5
C5—C6—C7	116.1 (3)	H2S2—C2S—H2S3	109.5
			
Zn2—O1—C1—C2	91.5 (3)	Zn2—O2—C15—C16	98.9 (3)
Zn1—O1—C1—C2	−139.4 (2)	Zn3—O2—C15—C16	−145.8 (2)
Zn2—O1—C1—C6	−88.3 (3)	Zn2—O2—C15—C20	−79.1 (3)
Zn1—O1—C1—C6	40.8 (3)	Zn3—O2—C15—C20	36.1 (3)
O1—C1—C2—C3	178.8 (3)	O2—C15—C16—C17	−176.3 (3)
C6—C1—C2—C3	−1.4 (4)	C20—C15—C16—C17	1.9 (4)
O1—C1—C2—Br1	−2.1 (4)	O2—C15—C16—Br3	0.3 (4)
C6—C1—C2—Br1	177.7 (2)	C20—C15—C16—Br3	178.48 (19)
C1—C2—C3—C4	1.4 (4)	C15—C16—C17—C18	−0.1 (4)
Br1—C2—C3—C4	−177.8 (2)	Br3—C16—C17—C18	−176.7 (2)
C2—C3—C4—C5	1.2 (4)	C16—C17—C18—C19	−0.9 (4)
C2—C3—C4—Br2	−178.9 (2)	C16—C17—C18—Br4	173.6 (2)
C3—C4—C5—C6	−3.6 (4)	C17—C18—C19—C20	0.0 (4)
Br2—C4—C5—C6	176.5 (2)	Br4—C18—C19—C20	−174.4 (2)
C4—C5—C6—C1	3.5 (4)	C18—C19—C20—C15	1.9 (4)
C4—C5—C6—C7	−178.2 (3)	C18—C19—C20—C21	176.9 (3)
O1—C1—C6—C5	178.7 (3)	O2—C15—C20—C19	175.4 (2)
C2—C1—C6—C5	−1.1 (4)	C16—C15—C20—C19	−2.7 (4)
O1—C1—C6—C7	0.6 (4)	O2—C15—C20—C21	0.7 (4)
C2—C1—C6—C7	−179.2 (3)	C16—C15—C20—C21	−177.5 (3)
C8—N1—C7—C6	−174.9 (3)	C22—N3—C21—C20	179.5 (3)
Zn1—N1—C7—C6	−3.0 (4)	Zn3—N3—C21—C20	−3.8 (4)
C5—C6—C7—N1	159.0 (3)	C19—C20—C21—N3	164.3 (3)
C1—C6—C7—N1	−22.8 (4)	C15—C20—C21—N3	−20.7 (4)
C7—N1—C8—C9	−150.8 (3)	C21—N3—C22—C23	119.6 (3)
Zn1—N1—C8—C9	36.7 (3)	Zn3—N3—C22—C23	−57.7 (3)
N1—C8—C9—C10	−76.7 (3)	N3—C22—C23—C24	81.3 (3)
C14—N2—C10—C11	0.0 (4)	C28—N4—C24—C25	−0.5 (4)
Zn1—N2—C10—C11	−171.6 (2)	Zn3—N4—C24—C25	178.0 (2)
C14—N2—C10—C9	−179.0 (3)	C28—N4—C24—C23	178.8 (3)
Zn1—N2—C10—C9	9.4 (4)	Zn3—N4—C24—C23	−2.6 (4)
C8—C9—C10—N2	50.5 (4)	C22—C23—C24—N4	−44.7 (4)
C8—C9—C10—C11	−128.5 (3)	C22—C23—C24—C25	134.6 (3)
N2—C10—C11—C12	1.4 (5)	N4—C24—C25—C26	−0.2 (4)
C9—C10—C11—C12	−179.6 (3)	C23—C24—C25—C26	−179.5 (3)
C10—C11—C12—C13	−1.2 (5)	C24—C25—C26—C27	1.0 (4)
C11—C12—C13—C14	−0.2 (5)	C25—C26—C27—C28	−1.1 (4)
C10—N2—C14—C13	−1.5 (4)	C24—N4—C28—C27	0.4 (4)
Zn1—N2—C14—C13	170.5 (2)	Zn3—N4—C28—C27	−178.2 (2)
C12—C13—C14—N2	1.6 (5)	C26—C27—C28—N4	0.4 (4)

Symmetry code: (i) −*x*+1, *y*, −*z*+1/2.

### Hydrogen-bond geometry (Å, º)

**Table:**

*D*—H···*A*	*D*—H	H···*A*	*D*···*A*	*D*—H···*A*
C9—H9*A*···Br1^i^	0.99	2.92	3.854 (3)	157
C9—H9*A*···O1^i^	0.99	2.60	3.317 (4)	129
C21—H21*A*···O14	0.95	2.57	3.080 (4)	114
C22—H22*A*···O14	0.99	2.58	3.099 (4)	113
C22—H22*B*···Br4^ii^	0.99	2.96	3.664 (3)	129
C23—H23*A*···O12^iii^	0.99	2.58	3.427 (4)	144
C28—H28*A*···N3^i^	0.95	2.60	3.236 (4)	125
C2*S*—H2*S*1···O11	0.98	2.60	3.556 (4)	165
C2*S*—H2*S*2···Br2	0.98	3.02	3.935 (4)	157
C2*S*—H2*S*2···Br4	0.98	3.04	3.561 (3)	115

Symmetry codes: (i) −*x*+1, *y*, −*z*+1/2; (ii) *x*, −*y*+1, *z*−1/2; (iii) −*x*+1/2, −*y*+3/2, −*z*.
